# Atypical Presentation of Bronchogenic Cyst in the Retroperitoneal Space

**DOI:** 10.7759/cureus.55029

**Published:** 2024-02-27

**Authors:** Karol Gostomczyk, Jędrzej Borowczak, Marek Zdrenka, Łukasz Szylberg

**Affiliations:** 1 Department of Obstetrics, Gynaecology and Oncology, Collegium Medicum Nicolaus Copernicus University, Bydgoszcz, POL; 2 Department of Pathology, Dr Jan Biziel Memorial University Hospital No. 2, Bydgoszcz, POL; 3 Department of Tumor Pathology and Pathomorphology, Prof. Franciszek Łukaszczyk Oncology Centre, Bydgoszcz, POL

**Keywords:** differential diagnosis, laparoscopic surgery, retroperitoneal nodule, adrenal lesion, cancer, bronchogenic cyst

## Abstract

Bronchogenic cysts, benign congenital malformations resulting from abnormal tracheobronchial tree budding, primarily manifest in the mediastinum, with retroperitoneal occurrence being exceedingly rare. Typically incidental findings on imaging, and their diagnosis pose challenges, particularly when malignancy is suspected.

We present a case involving a 55-year-old woman diagnosed with chronic back pain. Physical examination revealed a painful mass in the left renal region. Subsequent MRI identified a smooth mass in the left adrenal gland without infiltration of surrounding structures. Laparoscopic surgery successfully removed the lesion without complications. Pathomorphological examination confirmed a gelatinous-filled cyst, identified as a retroperitoneal bronchogenic cyst in the left adrenal gland.

Increasing reports of retroperitoneal bronchogenic cysts contribute to a better understanding of their characteristics, aiding preoperative diagnosis. However, given potential malignancy and definitive diagnosis through histopathological examination, surgical resection remains the preferred method

## Introduction

A bronchogenic cyst is a remnant of embryonic development that occurs mainly in the mediastinum. The causes of a bronchogenic cyst are not well understood [1]. Several mechanisms have been proposed, the most likely being abnormal growth of the upper gastrointestinal and respiratory tracts. Cysts are not caused by genetic or chromosomal disorders but are associated with abnormal or late budding of the embryonic ventral bud of the tracheobronchial tree [2-4]. Such a bud differentiates into a fluid-filled cyst connected to the respiratory system. The localization depends on the stage of development at which the malformation occurs. If the anomaly occurs very early in fetal development, the cyst may be found in the tracheobronchial tree [5-7]. If the anomaly occurs later during organogenesis, the cyst may be found in the lung parenchyma.

Bronchogenic cysts are most often found incidentally during an ultrasound scan or a computed tomography [6-9]. They usually cause no complications and are asymptomatic. If the cyst is large, it may exert pressure on surrounding structures and cause untypical symptoms such as dysphagia, hoarseness, non-localized pain, airway or oesophageal obstruction, hemoptysis, pneumothorax, pleuritis or even pneumonia if the cyst is infected [4-6].

Since they are associated with the trachea, they are also lined with ciliated pseudostratified columnar epithelium of the respiratory type. Sometimes there are areas of squamous metaplasia. Cartilage, smooth muscle, fibrous tissue, and glands are found within the cysts. Macroscopically, they are spherical, smooth, white or pinkish, single or multiple, often unilocular, 2 to 12 cm in diameter, filled with clear, proteinaceous fluid, or, rarely with hemorrhagic secretions [2-5]. Calcification of the cyst wall is very rare, as is communication with the bronchial tree [5].

Bronchogenic cysts are a diagnostic problem. They must be differentiated from branchial cleft cysts, pulmonary abscesses, hydatidosis, fungal diseases, tuberculosis, infected bullae, vascular malformations, neoplasms, cystic teratomas, bronchopulmonary sequestrations, oesophageal cysts and healed abscesses [7-9]. Diagnosis is difficult, especially when atelectasis or consolidation occurs in the vicinity of cysts, as they may be confused with tumors.

The case report was previously presented in the form of an oral presentation at the Vilnius Surgical Symposium on May 6, 2023, and is featured in the book of abstracts from this conference. Due to the considerable interest and its significance in the context of the differential diagnosis, this case has been transformed into a comprehensive article.

## Case presentation

A 55-year-old woman visited the Endocrinology Outpatient Clinic with complaints of discomfort in the left subcostal area and back pain that started a few months ago. Physical examination revealed no deviation from the norm: the patient had a fair complexion, no stretch mark, limbs proportional to the body, no maculopathy, ascites, or any Marfanoid and Cushinoid features. On physical examination, a palpable, painful nodule was found in the left renal region. MRI was performed in turbo spin echo (TSE) and gradient-recalled echo (GRE) sequences, obtaining T1-weighted, T2-weighted, T2-TIRM, and fat-suppressed images in three planes. Diffusion-weighted imaging (DWI), phase, and antiphase were performed in the transverse slices. Contrast was also administered. A smooth contoured mass was found in the left adrenal body, 19x13x32 mm. The mass did not infiltrate the surrounding structures, had a slightly lower signal in antiphase than in phase, and did not show contrast enhancement. The signal intensity factor was about 15%, indicating the ambiguous nature of the left adrenal nodule. The course of the examination was uncomplicated. No other pathologies were found on imaging.

Clinically, there was no obvious adrenal dysfunction, slightly increased urinary excretion of normetanephrine, or normal urinary excretion of metanephrine and methoxytyramine. No features of pheochromocytoma were found. TSH, FT3, FT4, and free cortisol were in the norm, while DHEA-S levels were slightly increased. 

**Figure 1:**
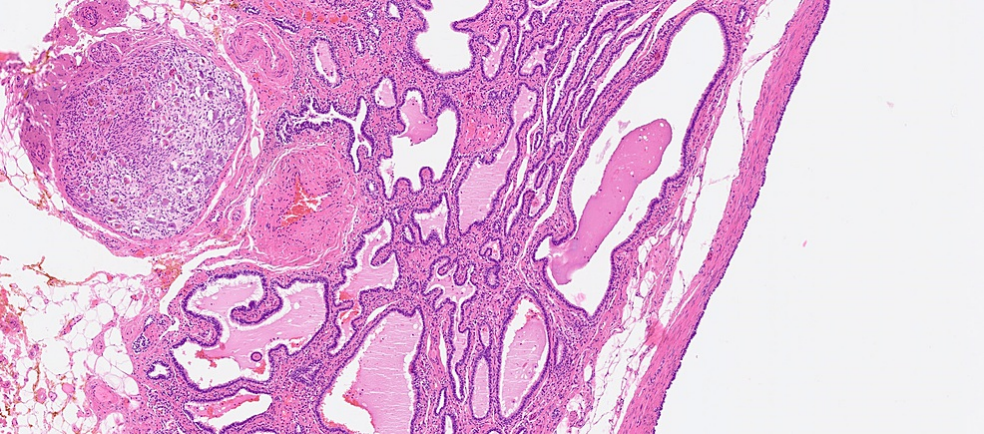
Bronchogenic cyst of the adrenal gland (magnification objective x4)

The patient was hospitalized in the Oncology Center, Department of Urology, and underwent a laparoscopic left adrenalectomy during which the mass was removed. Pathomorphological examination revealed a cyst filled with gelatinous masses measuring 25x12x12 mm (Figure 1).

**Figure 2:**
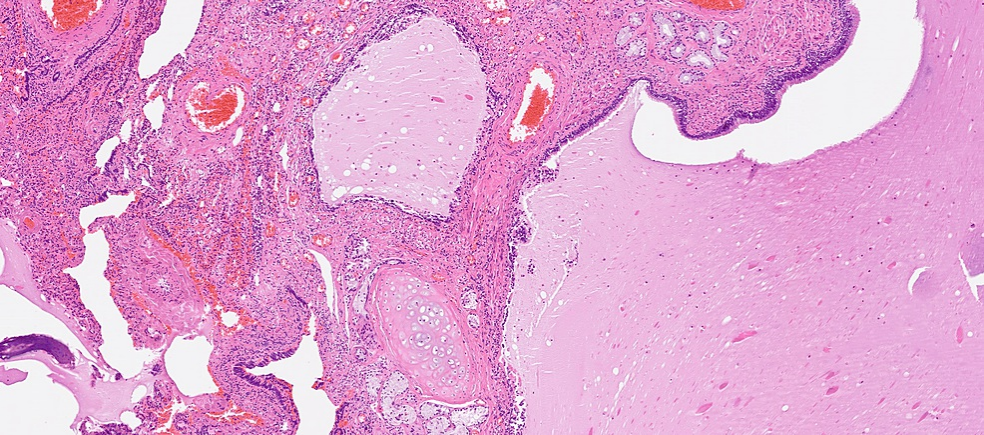
Bronchogenic cyst of the adrenal gland (magnification objective x4) The cyst is lined by respiratory-type epithelium and filled with serous material. Components of the cyst wall include seromucinous glands and hyaline cartilage. Chronic inflammation with epithelial denudation, hemorrhage, and fibrosis are also present.

Further histopathological diagnosis revealed a bronchogenic cyst located in the left adrenal gland (Figure 2).

The patient was discharged without complications. There was no evidence of recurrence at follow-up.

## Discussion

The cysts develop due to abnormal budding and pitching of the tracheobronchial tree [1]. As the connection to the tracheobronchial tree is lost, the foregut separates from the tracheal zone and migrates to an atypical location. For example, subdiaphragmatic cysts appear to migrate through the pericardioperitoneal canal connecting the thoracic and abdominal cavities [2-4]. However, bronchogenic cysts in the retroperitoneal region appear to be extremely rare. A review of the literature suggests that most retroperitoneal bronchogenic cysts are located close to the left adrenal gland [4-6].

While bronchogenic cysts can cause life-threatening compressive symptoms in children, they are often incidental radiological findings in adults [7]. In most cases, symptoms are secondary to infection or compression of adjacent organs. Fistulized bronchogenic cysts usually cause breathlessness, fever, and hemoptysis, whereas non-fistulized cysts are responsible for chest pain [8,9]. Because bronchogenic cysts are caused by the migration of lung buds, they are not metaplasia or ectopia of the lateral tissue. Therefore, the tissue adjacent to the retroperitoneum is pushed aside, causing back pain and upper abdominal pain [10]. Large cysts can obstruct the bronchi, causing air trapping and dyspnoea [11]. If close to the adrenal glands, the mass may affect the adrenal gland, causing pheochromocytoma-like symptoms, abdominal discomfort, and flank pain [12]. In this case, only urinary normetanephrine levels were elevated, with metanephrine and methoxytyramine levels within the norm. As the increase in normetanephrine is reported to be the first and only biochemical evidence of pheochromocytoma, the results prompted further intervention [13].

Imaging of retroperitoneal bronchogenic cysts remains challenging. Abdominal ultrasound is not preferred as it is limited by the poor accessibility of the adrenal gland. Instead, computed tomography (CT) is the tool of choice [14]. The cysts are usually well-circumscribed, spherical, or ovoid masses with variable fluid composition. Approximately 50% of them are fluid density (0-20 HU), but the degree of CT attenuation depends on the amount of internal proteinaceous content and may be similar to that of the surrounding mediastinal soft tissues [15,16]. On the other hand, magnetic resonance imaging (MRI) allows a better delineation of the anatomical relationships between the cyst and its surroundings. T2-weighted images show high signal intensity due to fluid content, but the intensity varies from low to high on T1-weighted images due to changing protein content [16,17]. There is often no enhancement after contrast injection. Wen et al. suggested that the characteristic imaging appearance of the retroperitoneal adrenal bronchogenic cyst (cartilaginous cyst wall, spindle-shaped peritoneal space, and fusiform shape) may facilitate its preoperative diagnosis [10].

However, the cyst can easily be misdiagnosed as a solid tumor because of its effect on adrenal hormone secretion. As it is virtually impossible to exclude neoplastic processes such as pheochromocytoma or adrenal cortical carcinoma, surgical diagnosis is recommended [10]. Bronchogenic cysts in other localisations can be managed and diagnosed by transbronchial or percutaneous aspiration under CT guidance. In these cases, follow-up may be considered, as these cysts have a tendency to grow, sometimes rapidly [18]. Such a protocol seems doubtful in the context of a suspected adrenal lesion. Therefore, surgical resection with subsequent histopathology to confirm the diagnosis remains essential.

Chung et al. recommended retroperitoneal laparoscopic surgery as an effective approach for the treatment of retroperitoneal bronchogenic cysts and was soon followed by other clinicians [19,20]. In our case, laparoscopic resection of the lesion relieved the patient's symptoms and allowed for good control of postoperative pain, no complications, and a relatively short inpatient stay, encouraging this approach as safe and cost-effective. In addition, the cyst is adherent to adjacent structures and its residual wall can be cauterized to prevent recurrence [10].

## Conclusions

The growing recognition of retroperitoneal bronchogenic cysts has improved preoperative diagnosis. Despite potential malignancy concerns and the need for histopathological confirmation, surgical resection remains the preferred treatment. Laparoscopic surgery stands out as the method of choice due to its minimally invasive nature, offering advantages such as reduced postoperative pain and quicker recovery. This approach, involving small incisions and specialized instruments, ensures precise cyst removal and facilitates thorough examination. Overall, the combination of laparoscopic surgery and histopathological assessment remains the gold standard for managing retroperitoneal bronchogenic cysts.
